# Impact of an Educational Intervention Implanted in a Neurological Intensive Care Unit on Rates of Infection Related to External Ventricular Drains

**DOI:** 10.1371/journal.pone.0050708

**Published:** 2013-02-04

**Authors:** Eduardo Fernandes Camacho, Ícaro Boszczowski, Maristela Pinheiro Freire, Fernando Campos Gomes Pinto, Thais Guimaraes, Manuel Jacobsen Teixeira, Silvia Figueiredo Costa

**Affiliations:** 1 Division of Infectious Diseases, Hospital das Clinicas, University of São Paulo School of Medicine, São Paulo, Brazil; 2 Division of Neurosurgery, Hospital das Clinicas, University of São Paulo School of Medicine, São Paulo, Brazil; 3 Medical Research Laboratory LIM-54, University of São Paulo School of Medicine, São Paulo, Brazil; University of Maryland, United States of America

## Abstract

**Background:**

Studies on the implantation of care routines showed reduction on EVD catheter-related infections rates; however zero tolerance is difficult to be achieved. The objective of this study was to assess the impact of an educational intervention on the maximal reduction on rates of EVD-related infections.

**Methodology/Principal Findings:**

The quasi-experimental (before-after intervention) study occurred in two phases: pre-intervention, from April 2007 to July 2008, and intervention, from August 2008 to July 2010. Patients were followed for 30 days after the removal of the EVD, and EVD-related infections were considered as only those with laboratorial confirmation in the CSF. Observations were made of the care of the EVD and compliance with Hygiene of the Hands (HH), a routine of care was drawn up, training was given, and intervention was made to reduce the time the EVD catheter remained in place.

**Results:**

during the study, 178 patients were submitted to 194 procedures, corresponding to 1217 EVD catheters-day. Gram-negative agents were identified in 71.4% of the infections during the pre-intervention period and in 60% during the intervention period. During the study, EVD-related infection rates were reduced from 9.5% to 4.8% per patient, from 8.8% to 4.4% per procedure, and the incidence density dropped from 14.0 to 6.9 infections per 1000 catheters-day (p = 0.027). The mortality reduced 12% (from 42% to 30%).

**Conclusions/Significance:**

During one year after the fourth intervention, no microbiologically identified infection was documented. In light of these results, educational intervention proved to be a useful tool in reducing these rates and showed also impact on mortality.

## Introduction

The incidence of External Ventricular Drain (EVD) related infection (ventriculitis/meningitis) may vary from less than 1% to more than 27% [1]. National Healthcare Safety Network (NHSN) proposes criteria for monitoring and reporting these infection rates [2]. Risk factors for EVD related infection include time that the catheter is in place, underlying disease, multiple infections, multiple catheter insertions, neurosurgery, cerebrospinal fluid (CSF) leakage, catheter exchanges, and the technique used for catheter insertion. Early detection of the EVD related infection is important for treatment success, which includes removing or changing the catheter [3]–[7]. Central Nervous System (CNS) infection rates are relatively low, although they present great morbidity and mortality [8]–[9]. Began et al. (1992) published a 21% mortality rate among patients during treatment with an EVD in place [10]. According to results from literature, various studies have succeeded in reducing rates of infection implementing a routine of care along with consistent educational intervention [11]–[13]. These studies achieved reductions from 40% to more than 70% in EVD catheter related infections [6]–[14].

The aim of our study was to evaluate the impact of an educational intervention implemented in a neurological intensive care unit (ICU) in reducing the rates of EVD-related infections. Before March 2008 there was no standardized routine of care for EVD catheters.

## Methods

### Setting

This study was conducted between April 2007 and July 2010 (40 months) in a neurology ICU with eleven-bed, at Hospital das Clinicas, a one thousand-bed Major Teaching Hospital affiliated to University of São Paulo, São Paulo, Brazil (HC-FMUSP). The study was approved by institutional review board. Neurology ICU at our institution is provided with a dedicated sink for hand hygiene (HH) at the entrance of the unit and four other sinks for HH are inside the unit, and a couple of sinks dedicated for medication preparation. All the sinks are provided with a dispenser containing chlorexidine gluconate soap (2%) for hand washing. There are 10 alcohol gel dispensers strategically distributed among the beds. Staff is composed by attending physicians and medical residents, nurses, nursing assistants and physical therapists (all referred to as healthcare professionals – HCP).

### Study Design

This is a quasi-experimental study (before-after intervention). Pre-intervention phase was between April 2007 and July 2008 (16 months) and intervention phase between August 2008 and July 2010 (24 months).

### Definitions

EVD-related infection was defined as meningitis or ventriculitis following an EVD insertion as criteria described by Horan et al. [2], patients had to meet both criteria 1 and 2 to be included i.e, only meningitis/ventriculitis with an isolated infectious agent. Infection was considered EVD related if a catheter was in place during diagnosis or up to 30 days after its removal.

### Outcomes

The primary outcome was defined as the incidence of microbiologic confirmed EVD-catheter related infection expressed as number of EVD-catheter infections per 1000 EVD-catheter-days. We have also presented these infections using other denominators, i.e, per patient and per procedure. Other outcomes analyzed were adhesion of seven items stated in a routine of care further described and they were hand hygiene (during the five moments as defined by World Health Organization), daily scalp hygiene, dressings (exchanging whenever soiled or loose), hair removal, availability of individual recipients to waste CSF collected from its reservoir at the distal site of the drainage system, closed drainage system in place, semirecumbent position.

We compared these outcomes during pre-intervention and post-intervention periods.

### Observations of EVD care and hand hygiene

Five observations of EVD care were made and one observation of HH; all observations were carried out by the researcher, on a weekly basis. At the time of observations, no healthcare worker was aware of it. The first observation of EVD care was performed from April to July 2007, before the introduction of the routine of care. The next four observations were performed in October 2008 (one month after the introduction of the routine of care), January 2009, October 2009 and January 2010 respectively. Observation of HH occurred in August 2009, one year after the first educational activity presenting the routine of EVD care.

### Surveillance of EVD related infection

Patients were followed up to 30 days after the catheter removal. Whenever the drainage system was replaced, a new surveillance form was initiated and the patient was followed up from the date of the catheter change, as long as this change was not related to a CNS infection. Surveillance was carried out by the researcher.

All patients undergone to EVD performed in the operating room and admitted to neurology ICU were included.

Patients who presented with brain injury with open fractures, CSF leakage, congenital hydrocephalus, presence of any active CNS infection at the time of the EVD insertion and EVD placement in any location other than the operating room were excluded.

### Interventions

#### Routine of EVD care

During the first three months of 2008, the infection control department along with nursing leadership and neurosurgeons, established a routine of EVD care.

The main points outlined by the routine statement were: HH before and after handling the EVD system; insertion of EVD in operating rooms; maintenance of semirecumbent position; use of clipper for whole scalp hair removal before cahteter insertion; skin preparation with chlorhexidine soap (2%) followed by alcoholic chlorhexidine (0.5%) before EVD insertion; administration of antibiotic prophylaxis using Cefuroxime 1.5 gram during anesthesia induction followed by 750 milligrams every four hours during the intraoperative phase, and 750 milligrams every eight hours in the postoperative phase up to 24 hours; performing a 5 cm tunneling from the EVD catheter insertion site to the trepanation point; verifying the presence of CSF leakage; performing daily dressings that included cleansing of the surgical incisions with saline solution, application of alcoholic chlorhexidine (0.5%), covering with dry sterile gauze, and wrapping of the head; use of maximal barrier protection during insertion and dressing (sterile drapes, gloves and gowns, head covers, and masks); performing of CSF culture only when infection is suspected; maintaining a closed EVD system in place; performing aseptic technique when handling the EVD system; do not attempt to unobstruct the catheter, if the system integrity is violated, it should be replaced; removing EVD as soon as possible; wasting CSF from the reservoir at the distal point of the system using an individual recipient for each patient; During the study period there was no change of materials trademarks such as catheters or dressings. Dressings were performed exclusively by neurosurgery medical residents.

### Educational intervention

Training sessions occurred in August 2008, February 2009 and 2010. These trainings consisted of forty minutes classes with an opened session for questions and answers and were focused on presenting the routine of EVD care and discussing the incidence of EVD related infections. Targeted population was neurosurgery medical residents, neurosurgeons and nursing staff from neurology ICU. Besides the exposition sessions outlined above other resources were used for training like handouts, posters and rounds. In July 2009, a special meeting took place with neurosurgeons focusing on reducing the length that EVD catheters were in place.

### Microbiology

Identification was performed by conventional biochemical tests and automated system Vitek® 2 (bioMérieux Vitek Inc. laboratory, Hazelwood, MO, USA). Susceptibility tests were performed by Vitek 2 and confirmed by E-test (AB Biodisk, Solna, Sweden) according to the clinical laboratory routine. Interpretation criteria were those from CLSI (Clinical and Laboratory Standards Institute) [14].

During the study period, there was no other research on the topic, and no catheters or dressing materials saturated with antibiotics or antiseptics were used. All dressings were handled exclusively by the neurosurgery medical residents.

### Statistics

SPSS for Windows 18.0 was used for data analysis. Categorical variables were presented as frequencies. Continous variables were presented as means, median, standard deviation and variation. Fisher's Exact Test was used when appropriate. Means were compared using Wilcoxon's Test. We considered significant values for *p*≤0.05. Infection rates were calculated as frequencies for patient and procedure. Incidence per 1000 catheters-day and EVD utilization rate were also obtained. Crude mortality was compared between pre-intervention and intervention periods.

## Results

**Figure 1:**
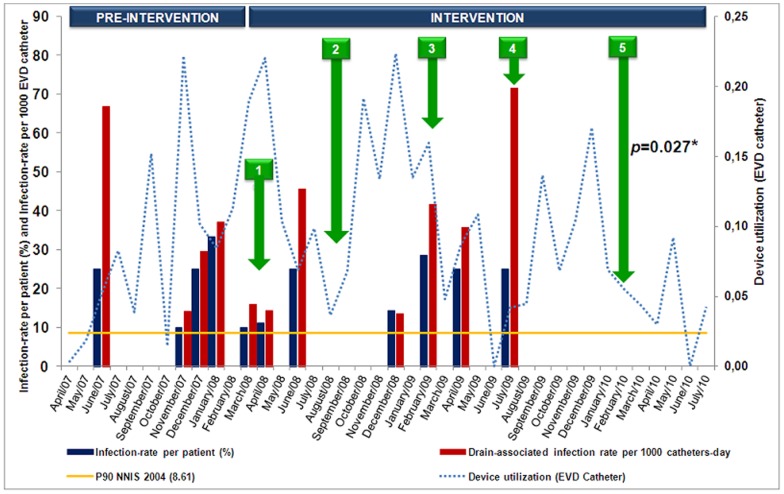
Distribution of indicators of EVD-related infections and the interventions carried out at the Neurological ICU of the Hospital das Clínicas – University of São Paulo School of Medicine during the period from April 2007 to July 2010. 1: Preparation of the routine of EVD care; 2,3 and 5: Expository classes; 4: Reduction in permanence of the EVD catheter; *: Time series (12 months without infection).

During the study, 178 patients undergone to 194 procedures, corresponding to 1217 catheters-day and 12 microbiologically confirmed EVD related infections. The mean age of the patients was 48 years old, and 62% of them were females. Crude mortality was 35%, 42% in the pre-intervention phase and 30% in the intervention phase (*p*<0.0001). Antibiotics were administered prophylactically in 80% of the procedures during the whole study period, 85% in the pre-intervention phase and 77% in the intervention phase (*p* = 0.2). Cefuroxime was administered in 82% of the cases. Timing of interventions is demonstrated in figure 1. One hundred and twenty one healthcare professionals (HCP) (92%), 94 HCP (72%) and 86 HCP (66%) were trained during the first, second and third interventions, respectively. During observations of EVD care in the pre-intervention period, it was noted that in 98% of the opportunities, dressings were not performed according to the stated routine of EVD care as well as 66% of scalp hygiene (Table 1). During intervention period, adhesion was enhanced (Table 1). Hand hygiene observation results are as follows: before and after handling the EVD system was 31% and 44%, respectively and before and after touching the patient or the surfaces around the patient was 19% and 76%, respectively (Table 2). EVD related infections during the study were reduced from 9.5% to 4.8% per patient (*p* = 0.4) from 9% to 4% per procedure (*p* = 0.2) and the incidence was reduced from 14 to 7 infections per 1000 catheters-day (*p* = 0.06). There was no microbiologically documented meningitis/ventriculitis during the 12 months following the fourth intervention. During the whole study period, eight infections were caused by Gram-negative rods and two infections yielded Gram-positive agents. Two polymicrobial infections were identified (Table 3). Figure 1 demonstrates how activities were distributed throughout the study.

**Table 1:** Distribution of the variables observed after the introduction of a routine of care.

OBSERVED VARIABLES	1st OBSERVATION	2nd OBSERVATION	3rd OBSERVATION	4th OBSERVATION
	May-July/2008	total N = 32	total N = 20	total N = 23
		October/2008	January/2009	October/2009
	A	A	A	A
	N (%)	N (%)	N (%)	N (%)
Daily scalp hygiene	46 (34)	31 (97)	20 (100)	22 (96)
Dressing	3 (2)	32 (100)	18 (90)	21 (91)
Hair removal (clipping) before insertion	not observed	32 (100)	19 (95)	20 (87)
Individual recipient to waste CSF*	129 (96)	32 (100)	20 (100)	23 (100)
Semirecumbent position 30 degrees	131 (97)	32 (100)	20 (100)	23 (100)
Closed system of drainage	131 (97)	32 (100)	20 (100)	23 (100)

* CSF – cerebrospinal fluid, A  =  ADEQUATE, N  =  number of observations.

**Table 2:** Observation of hand hygiene before and after handling of the EVD system and other handling of the patient during the intervention period.

VARIABLES	HAND HYGIENE
	OBSERVATION – AUGUST 2009
	A
	N/TOTAL (%)
Before handling the EVD system	39/125 (31.2)
After handling the EVD system	55/125 (44.0)
Before touching the patient	16/85 (18.8)
After touching the patient	65/85 (76.5)

A  =  ADEQUATE.

**Table 3:** Distribution of clinical and demographic data of 178 patients comparing the pre-intervention and intervention periods.

VARIABLES	PRE-INTERVENTION^1^	INTERVENTION^2^	*P* VALUE
**SAMPLE – N**			
Patient/Procedure	74/79	104/115	
Catheters-day	500	717	
**GENDER – N (%)**			
Male/Female	25 (32.4)/49 (67.6)	42 (40.4)/62 (59.6)	0.3716
**AGE – YEARS**			
Mean/Range	49/8–83	48/9–95	0.9390
**UNDERLYING DISEASE – N (%)**			
Hemorrhagic Stroke	7 (9.5)	16 (15.4)	0.2467
Ischemic Stroke	-	2 (1.9)	0.3689
Subaracnoid Hemorrhage	43 (58.1)	54 (51.9)	0.4154
Traumatic Brain Injury	4 (5.4)	3 (2.9)	0.3178
Brain Tumour	20 (27.0)	29 (27.9)	0.8998
**SURGICAL PROCEDURES – N (%)**			
Clipping Cerebral Aneurysm	6 (7.6)	16 (13.9)	0.1727
External Ventricular Drainage	39 (49.4)	59 (51.3)	0.7914
Hematoma Drainage	19 (24.1)	18 (15.7)	0.1445
Removal of Cerebral Tumour	15 (19.0)	22 (19.1)	0.9801
**ASA** ** **– N (%)**			
I	9 (11.4)	14 (12.2)	0.8689
II	21 (26.6)	38 (33.0)	0.3377
III	32 (40.5)	41 (35.7)	0.4940
IV	13 (16.5)	17 (14.8)	0.7521
V	4 (5.1)	5 (4.3)	0.5371
**ANTIBIOTIC PROPHYLAXIS – N (%)**			
Yes/No	67 (84.8)/12 (15.2)	89 (77.4)/26 (22.6)	0.2019
**ANTIBIOTIC – N (%)**			
Cefazolin	6 (9.0)	1 (1.1)	0.0250
Cefepime	2 (3.0)	-	0.1828
Cefoxitin	-	2 (2.2)	0.3239
Ceftriaxone	3 (4.5)	5 (5.6)	0.5250
Cefuroxime	52 (77.6)	77 (86.5)	0.1468
Meropenem	3 (4.5)	2 (2.2)	0.3678
Vancomycin	1 (1.5)	1 (1.1)	0.6760
Piperacillin/Tazobactam	-	1 (1.1)	0.5705
**SURGICAL TIME – MINUTES**			
Mean/Range	253/30–840	293/30–1050	0.5920
**CATHETERS – DAY**			
Mean/Range	6/<24h – 19	6/<24h – 21	0.9900
**LENGTH OF HOSPITALIZATION – DAYS**			
Mean/Range	32/<24h – 208	22/<24h – 72	0.1930
MORTALITY***			
N (%)	31 (41.8)	31 (29.8)	0.0962
**MICROORGANISMS N (%)**			
**GRAM-POSITIVE COCCI**	**1** (**14.3**)	**1** (**20.0**)	**0.7888**
* Enterococcus faecalis*	-	1 (20.0)	
* Staphylococcus aureus*	1 (14.3)	-	
**GRAM-NEGATIVE RODS**	**5** (**71.4**)	**3** (**60.0**)	**0.1056**
* Acinetobacter baumannii*	1 (14.3)	2 (40.0)	
* Pseudomonas aeruginosa*	-	1 (20.0)	
* Klebsiella pneumoniae*	2 (28.6)	-	
* Stenotrophomonas maltophilia*	1 (14.3)	-	
* Enterobacter cloacae*	1 (14.3)	-	
**POLYMICROBIAL**	**1 (14.3)**	**1 (20.0)**	**0.7888**
* Enterococcus sp* + *Acinetobacter baumannii*	-	1 (20.0)	
* Enterobacter cloacae + Klebsiella pneumoniae*	1 (14.3)	-	

** ASA  =  American Society of Anesthesiologists Score; *** Mortality: untill discharged or death.

Cathegorical variables were analyzed by *Chi*-square or Fisher's exact test and compare means by Wilcoxon test Variable statistical significant (p<0.05).

^1^Pre-intervention (16 months) ^2^Intervention (24 months).

## Discussion

Although the rates of EVD related infection during the study period were not significantly reduced, there was a shift towards reduction. Duration of the study might have limited to achieve lower rates.

It is important to note that during the 12 months following the fourth intervention there was no microbiologically documented meningitis/ventriculitis. Educational interventions and envolvement of healthcare workers (including the whole staff, i.e, nursing staff, neurosurgeons and medical residents) with the routine of care were the cornerstone of this interventional study. The results demonstrate that decreasing of infection EVD related meningitis/ventriculitis can be achieved using low cost educational intervention.

Although it is not possible to state that mortality was reduced due to the intervention there was a significant reduction during the intervention period (*p*<0.0001).

We related only microbiologically identified meningitis/ventriculitis because the diagnosis of this syndrome brings about difficulties in differentiating infection from aseptic meningitis after neurosurgery procedures. Of note, eight (11%) clinically defined meningitis/ventriculitis (without isolated agent in CSF) were identified during the pre-intervention and eleven (11%) (p = 0.90), during intervention phase, data not shown.

Korinek et al. (2005) [6] designed a protocol of care based on interventions and reduced the infection rates by 46.7%. In another study carried out by Dasic et al. (2006) [13], it was noted that the EVD related infection rates reduced 44.4% after interventions. Leverstein-van Hall et al. (2010) demonstrated that EVD-related infection rates were reduced by 55% and they proposed measures based on HCP training and preparing a routine of care for EVD handling [15]. Honda et al. (2010) performed a study during eight consecutive years aiming to assess the effect of three interventions on the incidence reduction of ventriculitis, and achieved a 76% decrease in the incidence of EVD-related infections (*p* = 0.066) [16]. In the present study, during three months before the introduction of a routine of EVD care consensus among infection control department, neurosurgeons and nursing staff, a survey on problems and difficulties in daily practice was conducted by observing the care given. As a result of this first observation, it was clear that in 66% of the opportunities, hygiene of the patient's scalp was not performed on a daily basis or even at all. Catheter insertion dressings were cared for in an unsafe manner, without antiseptics use. Dressings came lose easily due to the presence of sweat and natural oils of the patient's skin. During the study period, four other observations were made after the training sessions, which resulted in an improvement of care. During the last observation, performed in January 2010, adhesion to the recommended routine of care was 100%, except by HH which had 76.5% of adhesion by HCP after touching the patient or surfaces around. The protocol of the EVD routine of care was created driven by evidence based practices but consensus was achieved respecting some beliefs of HCP. Maximal barrier precautions to performing dressings by medical residents is not an evidence based practice but was implemented as a special request from neurosurgeons whose active participation was important to improve adhesion. In July 2009, a consensus on removal of the catheter when is no longer necessary was achieved. Nevertheless, there are controversies regarding the time an EVD catheter should remain in place. In 1984, Mayhall et al. recommended exchanging or removing the catheter every five days [17]. Holloway et al. (1996) revised the incidence of infection in 584 patients and the risk of infection increased during the first 10 days [18]. The rate of infection in patients whose catheters were substituted within five days was not lower than those of patients with catheters changed after longer intervals. For Korinek et al. (2005), the duration for more than five days was not a risk factor for EVD-related infection, and 67 patients had EVD catheters in place for more than 10 days (up to 42 days) with no infection [4]. Pfisterer et al. (2003), after evaluating 186 EVD procedures, concluded that length of catheterization was not a risk factor for infection [3]. On the other hand, for Rebuck et al. (2000), catheterization for more than five days was characterized as a risk of EVD-related infection [19]. Arabi et al. (2005) published that the risk of infection occurred predominantly during the first seven days that an EVD catheter is in place (*p* = 0.001) [3]. Hoefnagel et al. (2008) studied 228 patients with an inserted EVD, mean length of eight days, and concluded that the duration of catheter permanence was a risk factor for infection (*p*<0.0001) [20].

The weekly presence of the researcher in the ICU was important to reinforce the objectives proposed and to assure that the multi-professional team would not fail to reach the goals established. No microbiologically documented EVD related infection was reported up to one year after the fourth intervention. The fact that a single researcher observed adhesion and performed surveillance may constitute a limitation of our study. On the other hand, consistent criteria were used for the whole period and probably with low variation.

There was no significant difference in clinical and demographic data when the pre-intervention and intervention periods were compared, which was according to our expectation, showing that the population was homogeneous throughout the study. We had lower adherence to antimicrobial prophylaxis during intervention period, although not statistically significant. The use of antibiotic prophylaxis during EVD insertion is matter of debate [6], [8]. Lack of prophylactic antibiotics was not a risk factor for ventriculitis in one study [6] and did not achieve a reduction in the rate of infection [8]. In Hoefnagel et al. (2008), antibiotic prophylaxis was given only to 37.7% of patients, and there was no significant difference among receivers and non-receivers (p = 0.11) [20]. In this study, the presence of Gram-negative agents was very important (80%), and differs from the literature. Although there are few publications on this matter originated in South-American countries, there is an evident discrepancy in agents reported as causative of EVD-related ventriculitis. Additionally, infections caused by *Acinetobacter sp*, especially in critically ill patients, are endemic at our institution since 1993 when there was an outbreak of this agent [21]. According to Girão et al. (2008), the predominance of Gram-negative agents, especially *Pseudomonas aeruginosa*, *Acinetobacter baumannii*, and *Klebsiella pneumoniae* has been a problem in Latin American hospitals [22]. For Lietard et al. (2008), the *Staphylococcus aureus* agent was responsible for 75.8% of infections in neurosurgery [23]. Hoefnagel et al. (2008), found 59.3% of Gram-positive agents and 40.7% of Gram-negative agents [20]. Korinek et al. (2005) reported coagulase-negative *Staphylococcus* (50%) as the most frequent agent [6]. Chi et al. (2010) found 84% of Gram-negative agents such as *P. aeruginosa*, *A. baumannii*, and *Stenotrophomonas maltophilia* [24]. Krol et al. (2009) reported the emergence of *A. baumannii* as causative agent of meningitis, especially in emergency neurosurgery which had an EVD inserted [25].

This study presents several limitations as outlined above. However, these results showed that even in low resource countries it is possible to change trends of EVD related infection using low cost measures. Involvement of the staff was fundamental to shift EVD-related infection rates towards reduction.

## Conclusion

Educational intervention proved to be a useful tool towards reduction of EVD related infections. This intervention might have contributed to reduce mortality.
